# Effect of School Based Treatment on the Prevalence of Schistosomiasis in Endemic Area in Yemen

**Published:** 2013

**Authors:** A Abdulrab, A Salem, F Algobati, S Saleh, K Shibani, R Albuthigi

**Affiliations:** 1 Dept. of Internal Medicine, Faculty of Medicine &Health Sciences’ Sana'a University, Republic of Yemen; 2 Laboratory of Ministry of Health, Taiz Republic of Yemen; 3 Microbiology and Parasitology Taiz Laboratory, Republic of Yemen

**Keywords:** Schistosomiasis, School based programme, Prevalence, Yemen

## Abstract

**Background:**

Schistosomiasis and soil transmitted infection is a major health problem of children from rural areas of developing countries including Yemen. In an attempt to reduce this burden, the Ministry of Public Health and Population in Yemen established in 2002 a programme for Schistosomal, soil transmitted infection control that aimed to reduce morbidity and prevalence rates of Schistosomiasis, and Soil transmitted helminthes to less than 5% by 2015. The study was conducted to assess the current prevalence and intensity of schistosomal infection among schoolchildren in rural areas of the Taiz governorate after 6 years of running National Control Programme.

**Methods:**

Grade 3 schoolchildren from Shara'b Al-Raona district of Taiz Governorate were examined for infections with *Schistosoma mansoni* using Modified Kato–Katz method and *S. haematobium* applying filtration method in 1998/1999, comparing the prevalence and intensity of infection with base line study, which was done 6 years ago.

**Results:**

The *S. mansoni* prevalence in the study population was 31%, while the prevalence of *S. haematobium* was 18.6%. This result considerably is similar to the prevalence of base line study. The intensity of mild, moderate and severe infection for *S. mansoni* reached to 15.9%, 60.6% & 23.5% respectively. The severity of *S. haematobium* infection was 68.4%. It was exceptionally found that the prevalence of *S. haematobium* is increased.

**Conclusion:**

The high prevalence of schistosomiasis and low effectiveness of control programme against schistosomal infection in the study area demands consideration of alternative treatment approaches.

## Introduction

Schistosomiasis and soil-transmitted helminthiasis are important health problems in several developing countries with considerable morbidity and mortality (1). Epidemiological surveys indicated that schistosomiasis is widely distributed in The Republic of Yemen. Two main forms of human schistosomiasis or Bilharziasis exist in Yemen– Urinary schistosomiasis caused by *Schistosoma haematobium* infection and intestinal schistosomiasis caused by *S. mansoni* infection (2, 3).

The higher prevalence of infection found in rain –fed streams, and its associated channels, which are run in most localities in Taiz, Ibb, Thamar, Abyan and Hajjah governorates (4). The prevalence of schistosomiasis in school children in endemic areas of some governorates reached to 90% and the overall prevalence of both infection *S. mansoni* and *S. haematobium* were 32% (3–8). Schistosomiasis is very important public health problems in Yemen, with an estimated prevalence of 4-3 million infected people (9). This figure possibly represents a significant underestimation of the actual disease burden. Schistosomiasis was first documented in early fifty in Yemen (10). Its epidemiological pattern became evident only when a series of large-scale surveys were conducted in the early 1970s (4). Since then, schistosomiasis control has been a public health priority in Yemen and several activities have been carried out, but reduction of the prevalence and intensity of infection was not succeeded due to instability and interrupted of continuity of schistosomiasis control programme.

Taiz governorate is an endemic area for both *S. haematobium* and *S. mansoni* infections. Both species were reported by Hazza in 1982 in areas near Taiz city (8). The prevalence of *S. mansoni* among schoolchildren in two localities (Alselow and khadeer) reached to 59.8% and 44.5% respectively (8). National Schistosoma Control Programme was established in 2002 for control of Schistosomiasis and soil transmitted helminthes, depends on school-based strategy. Treatment against schistosomiasis was delivered using the WHO dose pole method for Schistosomiasis PZQ (600 mg tablets) with a single dose of albendazole (400 mg) was used against soil transmitted helminthes (1). The ultimate goal of the programme is to reduce morbidity and prevalence rates of schistosomiasis and soil transmitted helminthes to less than 5% by 2015.

This cross-sectional study was carried out to assess the current prevalence of schistosomiasis among schoolchildren in Shara'b Al-Rona district of Taiz Governorate after 6 years of running the programme.

## Materials and Methods

This cross sectional study was taken during 2008 in Shara'b Al-Rona district of Taiz governorate. Shara'b Al-Rona District lies 45 km North West Taiz consist of 22 villages had a population of 146,650 inhabitants. Most of the people are farmers working in the fields and continuing contact with schistosome-infected water. To facilitate the study and because the National Programme is school based programme, we listed all primary schools in Shara'b district, and 11 schools were randomly selected from this list. All children of the third-year class who were present on the day of the survey were included. The total number of children participated was 614.

### Parasitological diagnosis/Stool examination

A single stool sample was collected from each child. Modified Kato–Katz slides were prepared from each sample and examined on the same day to determine *S. mansoni* infections. Eggs were counted and individual intensity of infection was expressed as eggs per gram of faeces (e/g).

### Urine examination

One urine specimen was collected from each child to determine *S. haematobium* infection using the filtration method and microscopy. Generally, specimens were collected around noon (between approximately 10:00 and 13:00), 10 ml of urine was filtered through a nylon filter (pore size 12 µm, Millipore, United Kingdom) and the number of eggs counted under a microscope. Intensity of *S. haematobium* infection was expressed as number of eggs per 10 ml of urine (e/10 ml).

### Statistics

Data were double entered into Microsoft Excel 97 and corrected for data entry errors. Data analysis was carried out by in SPSS 10.0.5 for Windows.

## Results

Of the total of 614 children investigated, 423 (62.4%) were boys and 191 (47%) girls. The mean age of all children was 9 ±1.6 years: The range of ages was from 7 to 13 years. Urine samples were obtained from 612 pupils and stool samples from 590. The prevalence of schistosomiasis and intensity of infection in this study and base line survey is shown in (Table 1).

**Table 1:** Prevalence of schistosomiasis among schoolchildren in Shara'b Al-Rona district of Taiz, Yemen

Type of *Schistosoma*	Number Tested	Number positive (%)	Number Negative (%)	*P* Value
*Schistosoma mansoni*				
This study	590	183 (31.0)	407 ( 69.0)	0.09
Base line study	355	129 (36.3)	226 (63.7)	
*Schistosoma haematobium*				
This study	612	114 (18.6)	498 (81.4)	0.04
Base line study	361	49 (13.6)	312 (86.4)	

In the present study the overall prevalence of schistosome infection was (24.8%). *S. mansoni* eggs were identified in 183 stool samples (31%), whereas *S. haematobium* eggs were identified in 114 urine samples (18.6%). Double infection with both species was found in 12 cases. Comparing the prevalence of schistosome infection in this study and that of a base line survey we found no significance reduction of infection in school children in Shara'b Al- Nora district of Taiz Governorate. However the prevalence of S. *haematobium* is increased. The rate of mild and severe infections in the case of *S. mansoni* was 15.9% and 23.5%. Whereas, in *S. hematobium* it was 31.6% for mild Infection and 68.4% for severe infection. Moderate infection in *S. mansoni* was 60.6%. The comparison between the intensity of schistosoma infection at base line survey and this study is shown in Table 2.

**Table 2:** Intensity of *Schistosoma* infection among schoolchildren in Shara'b Al-Rona district of Taiz

Intensity of infection	This study Number (%)	Base line Study Number (%)
*Schistosoma mansoni*		
Mild 1-99 egg/gm	43 (15.9)	00 (0.0)
Moderate 100-399 egg/gm	111 (60.6)	81 (62.8)
Severe ≥400 egg/gm	29 (23.5)	48 (37.2)
*Schistosoma haematobium*		
Mild <50 egg/10ml	36 (31.6)	00 (00)
Severe >50 egg/10ml	78 (68.4)	49 (100)

## Discussion

Two species of schistosome infection were identified in the study population. The combined prevalence of schistosomiasis in this study was 24.8% more or less similar to baseline study with little deferent despite the passage of 6 years which, indicates that transmission and epidemiology appear not to have changed much. However, prevalence of *S. haematobium* is higher than rates reported previously from Shara'b Al-Rona district (18.3%, vs., 13.0%). Our results are in line with the recent study done by Al-Shamiri A.H, et al. in 5 districts of Taiz governorate, they found the overall prevalence rate was 20.76% for *S. mansoni* and 7.41% for *S. haematobium* (11). Similar results had been reported from different localities in Yemen (7, 12, 13).

In Assahul valley of lbb governorates the prevalence of *S. mansoni* was found to be 35% coincides with our study (6). However, the reduction in prevalence and intensity of infection was previously reported by several studies from different localities in Yemen, using an annual treatment strategy and health education with varying results (14–17).

One of the factors likely to have contributed to the high stable prevalence of schistosomal infection in Shara'b Al-Raona and may be in the other similar districts in Taiz is the low treatment coverage over a relatively long space of time by the control programme. It was proofed that a high treatment coverage rate is instrumental in controlling schistosomiasis transmission (18). Studies from different areas in Africa conformed that the repeated mass treatment (with praziquantel) had significant reduction in the prevalence and intensity of infections (19–25) (Table 3).

**Table 3:** Effectiveness of repeated mass treatment on the prevalence (%) of schistosomiasis

Study citation	Country Location	Base line Prevalence		Prevalence after 4 weeks		Prevalence after 8 weeks
*Schistosoma mansoni*			**First round treatment**		**Second round treatment**	
Barakat (19), 2010	Egypt	44		29		26
Utzinger (20), 2000	Cote d'Ivoire	77		20		11
Black (21), 2009	Kenya	83		34		-
Picquet (22), 1998	Senegal	87		29		12
*Schistosoma haematobium*						
Midzi (23), 2008	Zimbabwe	51		6		8
Mduluza (24), 2001	Zimbabwe	52		3		-
Sacko (25), 2009	Mali	95		1		1

It is clear from this table that treatment with two doses of praziquantel were significantly reduced the prevalence of infection in both schistosomiasis in different areas of varying intensity of infection.

On the other hands, long-term effectiveness of mass treatment was questionable. Several reports had been done to determine the prevalence of schistosome infections before and several years after one mass treatment using praziquantel the prevalence of schistosomiasis in schoolchildren remained nearly constant (26–28) (Table 4).

**Table 4:** Long-term outcomes of school-based treatment for control of schistosomiasis

Place, author's and years of Examination	Prevalence of schistosomiasis	
	*Schistosoma haematobium* %	*Schistosoma mansoni* %
This study		
Before mass treatment 2002	13.6	36.3
After mass treatment 2008	18.6	31.0
Mali (Clements et al.) (26)		
Before mass treatment (1989)	25.7	7.4
After mass treatment (2006)	38.3	6.7
Mali (Landoure A et al.) (27)		
Before mass treatment (2004)	88.0	17.0
After mass treatment (2010)	61.0	12.0
Tanzania (Poggensee G et al.) (28)		
Before mass treatment 1996	36.7	22.9
After mass treatment 2002	33.6	00.7

As reinfection patterns normally vary from one area to another, it was thought that a single schedule treatment might not be entirely successful to reduce prevalence of infection. This trend is very clear from the various studies shown in table (4). The prevalence of the infection was near to base line or slightly higher than before intervention. Regarding the intensity of infection found to be threefold higher at the end of the study. This reinfection during the end of the study was attributed to the increased transmission (26–28).

In our study, 68.4% and 23.5% of children were heavily infected with *S. haematobium* and *S. mansoni* respectively. This could be attributed to reinfection or to redundant of programme to control infection as it is adopted to treat children that enrolled in schools leaving the other spreading infection and transmission in the community. In a school-based sch-istosomiasis control programme in Egypt it was reported that 80% of infected girls were not treated because they were not enrolled in schools and prevalence of infections with *S. haematobium* and *S. mansoni* were higher in non-enrolled school-age children (29). In Kenya it was noted that in some schools the prevalence of hematuria was not affected, or actually increased, after the first year of intervention due to enrolment of untreated children after mass treatment was given (30). MAM Nagi (2005) stated that “the outcomes of school-based programmes seem to be easily influenced by the pool of untreated children outside the school” (16). It was reported that school-based programmes would treat only between 31% and 50% of the infected persons and eliminate only 27% of the total number of *S. mansoni* eggs excreted (31).

Moreover, it was noted in our study area the treatment was delivered only during the collection of base line data and there was no continuous activity had been done after that. These important factors together may have played a role in the maintaining the transmission of infection. Therefore, continuous monitoring and activities should be considered when implementing a National Control Programme in area of high prevalence of schistosomiasis to reduce the prevalence, intensity and consequence of morbidity (32). Ultrasonography study in Yemeni patients revealed that bladder abnormalities (thickness, hyper-echogenicity and polypoid lesions) were found in 82% of patients and upper tract lesions in 54% of patients of *S. haematobium* infection (33). Squamous cell carcinoma is well known as a complication of *S. haematobium*, however urinary schistosomiasis with simultaneous bladder squamous cell carcinoma and transitional cell carcinoma has been reported from Iran (34). Nevertheless, interrupted of drug distribution not only contributed to increase the prevalence and intensity of S. infection, but also could potentiate rebound of schistosomal infection with complications (35).

Elimination of infection only can be achieved by a combination of health education, improvements in sanitation, population chemotherapy and general development (29, 35). Repeated interventions are thus necessary to ensure continuity of morbidity control. Financial, logistic and organizational limitations are major constraints to the long-term sustainability of such a strategy (29, 36). The available health infrastructure should be enrolled in the control strategy and be integrated into basic health services, enabling it to be sustained and making it affordable for the public.

## Conclusion

The unchanged level of schistosomiasis infection after 6 years of establishing Schistosomal Control Programme; demands consideration of alternative treatment approaches. Our study also suggests that monitoring and evaluation is a crucial component of the national control programme for treatment and control strategy according to national and local epidemiological conditions.
